# Assessing Causal Associations of Atopic Dermatitis With Heart Failure and Other Cardiovascular Outcomes: A Mendelian Randomization Study

**DOI:** 10.3389/fcvm.2022.868850

**Published:** 2022-06-15

**Authors:** Heng Chen, Chengui Zhuo, Liangrong Zheng

**Affiliations:** ^1^Department of Cardiology, The First Affiliated Hospital, College of Medicine, Zhejiang University, Hangzhou, China; ^2^Department of Cardiology, Taizhou Central Hospital (Taizhou University Hospital), Taizhou, China

**Keywords:** Mendelian randomization, causal association, atopic dermatitis, heart failure, cardiovascular diseases

## Abstract

**Background and Aims:**

Observational epidemiological studies have suggested that atopic dermatitis (AD) was associated with an increased risk of cardiovascular diseases (CVDs). However, causality remains to be established. In the present study, Mendelian randomization (MR) analyses were used to evaluate whether AD and CVDs are causally associated.

**Methods:**

This study was based on summary statistics of genome-wide association studies (GWASs) for a set of cardiovascular outcomes including heart failure (HF), coronary artery disease (CAD), myocardial infarction (MI), atrial fibrillation (AF), stroke, and stroke subtypes. A total of 19 independent single nucleotide polymorphisms associated with AD were identified at a genome-wide significance threshold (*P* < 5 × 10^−8^) based on a large GWAS meta-analysis. MR estimates were pooled using the inverse variance weighted method. Complementary analyses further evaluated the robustness of the results.

**Results:**

Genetically determined AD was causally associated with HF [odds ratio (OR), 1.07; 95% confidence interval (CI), 1.03–1.10; *P* = 1.11 × 10^−4^]. However, there was no causal association between AD and the risk of AF, CAD, MI, stroke, and stroke subtypes. Complementary analyses returned similar results. No horizontal pleiotropy was found.

**Conclusion:**

This MR study provided evidence to support that AD exerted an effect contributing to HF. No significant associations were found for other cardiovascular outcomes. The study suggested that prevention and early diagnosis of AD may help prevent HF. Improved awareness of these associations is warranted for better management of CVDs in the future.

## Introduction

Cardiovascular diseases (CVDs) remain the leading cause of morbidity and mortality globally (1). The most common clinical manifestations of CVDs include heart failure (HF), coronary artery disease (CAD), cerebrovascular disease and atrial fibrillation (AF). Although the nature of CVDs is complex and not fully understood, common risk factors, such as hypertension (2), obesity (3), hyperlipidemia (4), and smoking (5), have been recognized as contributing to the early management of CVDs. In addition, observational studies have further suggested that atopic dermatitis (AD) is a potential risk factor for CVDs (6).

AD (also known as atopic eczema) is a common inflammatory skin disease, which typically begins in childhood and affects 5–10% of adults (7). A meta-analysis of longitudinal cohort studies showed that participants with AD experienced an increased risk of HF, myocardial infarction (MI), and stroke compared to those without AD (6). Cross-sectional studies have further revealed that CVDs were more prevalent in patients with AD (8). In addition, the risk of CVDs appeared to increase in patients with more severe and recalcitrant AD (6). However, the results from observational studies are inconclusive regarding the causal effects of AD on CVDs given to biases such as residual confounding, misclassification, and reverse causality (9). In addition, observational studies may not reveal the association of life-long exposure to AD with CVDs risk.

Recognition of the causal links between genetic predisposition to AD and CVDs may provide new avenues for the management of CVDs. In the present study, we conducted a Mendelian randomization (MR) study to clarify the causal associations between AD and CVDs. MR is a powerful approach to evaluate the causal associations between exposures and outcomes by using genetic variants as instrumental variables (IVs) (10). Given the genetic variants that are randomly assorted and fixed at conception, the approach diminishes the influence of potential confounding factors and reverse causality, thereby strengthening the causal inference (10). Here, we sought to explore the causality in the association of genetically determined AD with CVDs (CAD, MI, AF, HF, and stroke).

## Methods

### Study Design

A two-sample MR approach was employed to investigate the potential causal effects of AD as exposure on CVDs as outcome traits. The study was based on the following three key assumptions: (1) IVs are robustly associated with AD; (2) IVs must not be associated with potential confounders; and (3) IVs should affect the CVDs exclusively through AD (11). Ethics approval and informed consent were provided in all original researches included in the public genome-wide association studies (GWASs).

### Data Sources for HF and Other Cardiovascular Outcomes

Detailed information on all the summary statistics used in the MR study is provided in Table 1. There was no sample overlap between GWAS meta-analysis for AD and CVDs outcomes (Supplementary Table 1).

**Table 1:** Detailed information of studies and datasets used for analyses.

**Phenotype**	**Data source**	**Cases**	**Controls**	**Population**
Atopic dermatitis	EAGLE (12)	21,399	95,464	European
Heart failure	HERMES (13)	47,309	930,014	European
Heart failure	The UK Biobank Heart Failure GWAS (14)	6,504	387,652	European
Atrial fibrillation	Nielsen et al. (15)	60,620	970,216	European
Coronary artery disease	CARDIoGRAMplusC4D (16)	60,801	123,504	77% European
Myocardial infarction	CARDIoGRAMplusC4D (16)	43,676	128,199	77% European
Any stroke	MEGASTROKE (17)	40,585	406,111	European
Ischemic stroke	MEGASTROKE (17)	34,217	406,111	European
Large artery stroke	MEGASTROKE (17)	4,373	146,392	European
Small vessel stroke	MEGASTROKE (17)	5,386	192,662	European
Cardioembolic stroke	MEGASTROKE (17)	7,193	204,570	European

*EAGLE, EArly Genetics & Lifecourse Epidemiology; HERMES, Heart Failure Molecular Epidemiology for Therapeutic Targets; CARDIoGRAMplusC4D, Coronary ARtery DIsease Genome-wide Replication and Meta-analysis plus The Coronary Artery Disease Genetics*.

Summary statistics for the association between IVs and HF was obtained from two large GWAS datasets: (1) the Heart Failure Molecular Epidemiology for Therapeutic Targets (HERMES) Consortium (47,309 cases; 930,014 controls) (13) and (2) the UK Biobank Heart Failure GWAS (6,504 cases; 387,652 controls) (14). Participants in both studies were of European ancestry. In the HERMES consortium, patients with a clinical diagnosis of HF of any etiology were identified as cases, and there were no inclusion criteria based on the left ventricular ejection fraction (13). In the UK Biobank Heart Failure GWAS, HF was identified by the presence of self-reported “HF/pulmonary edema” or “cardiomyopathy” at any visit or an International Classification of Diseases (ICD) code for HF (ICD-10 or ICD-9) which indicates heart/ventricular failure or cardiomyopathy of any cause (14).

Summary-level data on AF were obtained from the largest published GWAS meta-analysis that included up to 60,620 cases and 970,216 controls of European ancestry from the Nord-Trøndelag Health Study, deCODE, the Michigan Genomics Initiative, DiscovEHR, UK Biobank, and the AFGen Consortium (15). Cases included participants with paroxysmal or permanent AF or atrial flutter (15). For CAD and MI, summary-level data were extracted from the Coronary Artery Disease Genome-Wide Replication and Meta-analysis plus the Coronary Artery Disease Genetics (CARDIoGRAMplusC4D) Consortium's 1,000 genomes-based genome-wide association meta-analysis (60,620 cases; 970,216 controls) (16). Subjects with an inclusive diagnosis of CAD (MI, acute coronary syndrome, chronic stable angina, or coronary stenosis of >50%) were defined as cases (16). Summary statistics for any stroke (AS), ischemic stroke (IS), and IS subtypes in patients of European ancestry were extracted from the MEGASTROKE consortium which included 40,585 cases and 406,111 controls (17). Stroke was defined as focal (or global) signs of rapidly developing brain dysfunction lasting >24 h or resulting in death without an apparent cause other than a vascular source (17). Based on the Trial of Org 10,172 in Acute Stroke Treatment criteria (18), IS was further classified as large artery stroke (LAS), cardioembolic stroke (CES), and small vessel stroke (SVS).

### IVs Selection

Single-nucleotide polymorphisms (SNPs) associated with AD were identified from the largest GWAS meta-analysis conducted by the EArly Genetics & Lifecourse Epidemiology (EAGLE) eczema consortium (12), with a total of 21,399 cases (diagnosed by physicians or self-reported) and 95,464 controls of European descent (12). A total of 21 SNPs were extracted from the study with genome-wide significance (*P* < 5 × 10^−8^) (Supplementary Table 2). Then we pruned one SNP (rs12730935) for linkage disequilibrium (r^2^ <0.001; region size, 10,000 kb). A cut-off for minor allele frequency was set to >1%, and no SNP was removed in this step. To meet the second key assumption that IVs must not be associated with potential confounders, we searched the PhenoScanner database and removed rs4713555 because it was associated with potential confounders, including “self-reported hyperthyroidism or thyrotoxicosis” and “self-reported type 1 diabetes” (*P* < 5 × 10^−8^) (19). Finally, 19 SNPs were selected as IVs for the MR analyses (Supplementary Table 2). In addition, F-statistics were calculated using the following formula: F = R^2^ × (N – 2)/(1 – R^2^) (20), where R^2^ refers to the proportion of the variance explained by IVs [calculated using the method described previously (21)], and *n* represents the sample size. Higher F statistic indicates a lower risk of weak IV bias. All of the SNPs were available in the outcome datasets except for the UK Biobank Heart Failure GWAS (rs12188917 and rs6419573 were not available). Therefore, we searched an online website (http://snipa.helmholtz-muenchen.de/snipa3/) and found two proxy-SNPs (r^2^ > 0.8) to replace them (rs3091307 for rs12188917 and rs1035127 for rs6419573, respectively).

### Mendelian Randomization Analyses

SNP-AD and SNP-CVDs associations were combined as a ratio to estimate the causal effects. The ratio estimates were calculated using the inverse variance-weighted (IVW) method in the random-effects model. The IVW analysis uses the reciprocal of the result variance as the fitting weight, providing the highest statistical power if the MR assumptions are met.

A set of complementary analyses were carried out to account for different patterns of pleiotropy and evaluate the robustness of the results. The weighted median method resulted in an unbiased estimate of causality even when up to 50% of the information in the analysis comes from invalid IVs (22). The MR-Egger regression method is more conservative and gives a consistent estimate when all IVs are invalid IVs (23).

Bias introduced by pleiotropic IVs may affect causal estimates. Thus, several methods were used to identify and address potential pleiotropy. First, the Cochran Q- heterogeneity test (24) was conducted to detect the presence of heterogeneity. A Cochran's Q *P*-value of > 0.05 was considered to indicate a low level of heterogeneity, suggesting that estimates between IVs vary by random chance and lack pleiotropic effects. Second, the MR-PRESSO method was employed to identify any potential horizontal pleiotropic outliers that may bias the results (25). Third, the intercept of the MR-Egger regression was used to assess horizontal pleiotropy present in the data averaged across the IVs. A zero-intercept *P*-value for MR-Egger of > 0.05 indicated a minor pleiotropic bias (26). Finally, as mentioned above, all SNPs in the PhenoScanner database were searched to exclude any SNP that was associated (*P* < 5.0 × 10^−8^) with risk factors for CVDs (19).

Due to multiple testing, a two-sided *P*-value of < 0.0056 (=0.05/9 outcomes) was set as the threshold for significance. Statistical power was calculated using a web-based tool (http://cnsgenomics.com/shiny/mRnd/) (27). The power estimates for AD were calculated based on a type 1 error of 5% (Supplementary Table 3). Scatter plots depicting the significant associations were also generated. All analyses were performed using the open-source statistical software R (version 4.1.0) with R packages including TwoSampleMR (28), MendelianRandomization (29), and MR-PRESSO (25).

## Results

Characteristics of the genetic associations of IVs with AD and CVD outcomes are shown in Supplementary Table 2. The included 19 SNPs were estimated to account for 5.63% of the phenotypic variability of AD. All of the SNPs had F-statistics higher than the threshold of 10, indicating a minor weak instrument bias in the present study.

**Figure 1:**
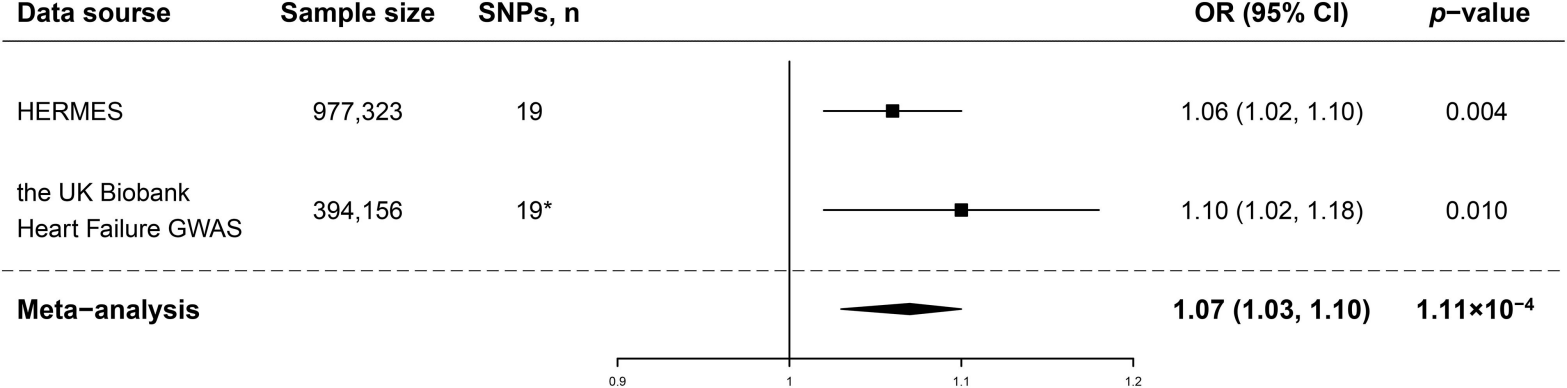
Associations of genetic predisposition to atopic dermatitis with risk of heart failure (HF). SNPs, single-nucleotide polymorphisms; OR, odds ratio; CI, confidence interval; HERMES, Heart Failure Molecular Epidemiology for Therapeutic Targets. Results were obtained from the inverse variance-weighted method in the random-effects model. *rs12188917 and rs6419573 were not available in the outcome dataset; rs3091307 and rs1035127 were found to replace them, respectively.

**Figure 2:**
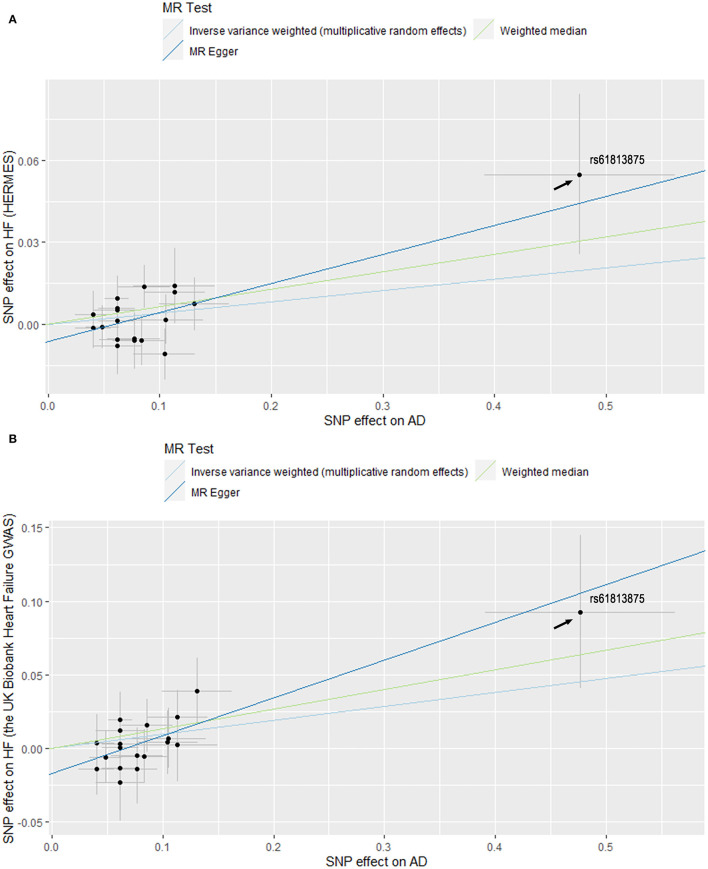
Scatter plots of MR estimates of genetic predisposition to atopic dermatitis (AD) on heart failure (HF) using datasets from HERMES **(A)** and the UK Biobank Heart Failure GWAS **(B)**. Standard errors were denoted by horizontal and vertical lines. The slope of each line corresponds to the estimated MR effect using different methods. SNPs, single-nucleotide polymorphisms; HERMES, Heart Failure Molecular Epidemiology for Therapeutic Targets.

In the standard IVW analyses, genetic predisposition to AD was causally associated with a higher risk of HF in the HERMES (odds ratio (OR), 1.06; 95% confidence interval (CI), 1.02–1.10; *P* = 0.004; Figures 1, 2). The association of AD with HF risk was replicated in the UK Biobank Heart Failure GWAS (OR, 1.10; 95% CI, 1.02, 1.18; *P* = 0.010; Figures 1, 2) and persisted in the meta-analysis (OR, 1.07; 95% CI, 1.03, 1.10; *P* = 1.11 × 10^−4^; Figures 1, 2). In the complementary analyses, the causal effect of AD on HF remained consistent in the weighted median and MR-Egger methods (Table 2).

**Table 2:** Associations of genetic predisposition to atopic dermatitis with risk of heart failure in complementary Mendelian randomization analyses.

**Data source**	**Sample size**	**SNPs, n**	**Methods**	**OR (95% CI)**	***P*-value**
HERMES	977,323	19	Weighted median	1.09 (1.01, 1.17)	0.026
			MR-Egger	1.13 (1.01, 1.26)	0.037
The UK biobank heart failure GWAS	394,156	19^*^	Weighted median	1.14 (1.00, 1.31)	0.048
			MR-Egger	1.29 (1.05, 1.59)	0.027

*SNPs, single-nucleotide polymorphisms; OR, odds ratio; CI, confidence interval; HERMES, Heart Failure Molecular Epidemiology for Therapeutic Targets*.

* *rs12188917 and rs6419573 were not available in the outcome dataset; rs3091307 and rs1035127 were found to replace them, respectively*.

For other CVDs (AF, CAD, MI, AS, IS, LAS, CES, and SVS), patients with AD may not have an increased risk of these outcomes compared to the controls (Supplementary Figure 1). The null effects of AD on these diseases were further supported by the complementary analyses results (Supplementary Figure 1).

For all considered outcomes, the MR-Egger intercept was close to zero (P_intercept_ > 0.05), indicating a low level of directional pleiotropy in the present study (Supplementary Table 4). No evidence of heterogeneity was found except for AS (${\text{P}}_{\text{Cochra}{\text{n}}^{\text{′}}\text{sQ}}$ = 0.03; Supplementary Table 4). Importantly, the MR-PRESSO method did not detect any horizontal pleiotropic outliers in all reported results (Supplementary Table 4).

Furthermore, rs61813875 was found to be different from the rest of the SNPs (Figure 2). It had the largest association with AD and HF. Therefore, a sensitivity analysis without this SNP was carried out to test the robustness of the results. The standard IVW analyses showed that the total effect of AD on HF remained significant after excluding rs61813875 (OR, 1.05; 95% CI, 1.01, 1.09; *P* = 0.009; Supplementary Figure 2). The association remained directionally consistent in the complementary analyses, albeit with wider CIs (Supplementary Table 5).

## Discussion

In the present study, two-sample MR analyses demonstrated that genetic predisposition to AD is causally associated with a higher risk of HF. However, no significant associations between AD and AF, CAD, MI, stroke, and stroke subtypes were found. The results were consistent and robust in complementary analyses using the weighted median and MR-Egger methods. To the best of our knowledge, this is the first MR study to explore the causal associations between AD and CVDs.

The observation that AD may be associated with CVDs and their risk factors (8, 30, 31) has prompted investigators to look for more evidence to confirm this association. The present results regarding the AD-HF association were consistent with a previous cohort study that identified AD as an independent risk factor for HF (hazard ratio (HR) 1.46, 95% CI, 1.10, 1.93) (32). The positive association was further supported by a large cohort study which reported that patients with AD experienced a higher risk of HF (HR 1.19, 99% CI, 1.09, 1.30) independent of common cardiovascular risk factors (33). Mechanistic work suggested that AD may exert a procoagulant effect (34), contributing to CAD and MI and ultimately leading to HF. However, the associations between AD and CAD or MI were inconsistent in the literature. An analysis of three cross-sectional surveys in U.S. populations found a 2.5-fold increased prevalence of CAD in patients with AD compared to their controls. The associations remained significant after adjustment for body mass index, smoking, alcohol consumption, and physical activity (8). Conversely, studies from Canada, Germany, Denmark, and the U.S.A. found either no associations or negative associations between AD and MI (35–38). In addition, the present MR study suggested that AD was not causally associated with MI, CAD, or AF, leaving the question of how AD can lead to HF open.

Disrupted barrier function was commonly observed in affected and unaffected skin of atopic subjects (39). This condition does not only lead to typical skin dryness and physical damage from scratching, but also induces inflammatory responses and overproduction of reactive oxygen species (39, 40). These pathophysiology drivers can promote and interact with others. Also, chronic skin inflammation and oxidative stress can cause other cutaneous and non-cutaneous diseases (40–43). As inflammation and oxidative stress are also involved in the pathogenesis of HF (44, 45), these conditions are likely to mediate the causal effect of AD on HF. On the other hand, clinical data showed that hypertension was a common comorbidity of AD (46). Prolonged pressure overload-induced by hypertension can lead to pathological cardiac hypertrophy and progression to HF (47). Therefore, hypertension is likely to, at least in part, explain the observed association between AD and HF.

The correlation between AD and stroke remains inconclusive so far. A large cohort study showed AD to be associated with an increased risk of stroke (HR 1.10; 99% CI, 1.02, 1.19) (33). However, a meta-analysis including eight related studies reported that AD may not be a relevant risk factor for stroke (OR 1.15; 95% CI 0.95–1.39) (48). The results of non-significant associations were in agreement with those obtained in the present MR study. Genetic liability to AD was not causally associated with the risk of stroke or its subtypes. The positive results observed in some observational studies may be attributed to residual confounders or reverse causality.

Anti-inflammatory therapy and barrier function improvement are recommended for the prevention and treatment of AD (39). Therapies like anti-hypertension and lipid-lowering and lifestyle changes, such as smoking cessation and appropriate sleep duration, can improve the management of HF and other CVDs (49–52).

The present study had several notable strengths. First, the MR approach was less susceptible to potential confounding factors and reverse causation, thus reinforcing the causal inference. Second, complementary analyses returned effect estimates similar to those obtained in the main analysis, thus strengthening the evidence. Third, a meta-analysis combining the results from two GWASs increased the power to detect an AD-HF association. Finally, the bias introduced by population structure was less likely to affect the results as the analyses were restricted to individuals of European ancestry.

However, there were several limitations to consider. First, the population in the present study, as mentioned above, was restricted to European ancestry. Therefore, the generalizability of study conclusions might be limited. Future studies on other populations are therefore warranted. Second, some psychiatric traits (such as major depressive disorder (MDD) and neuroticism) shared genetic liability with AD and CVDs (53–55). Specifically, there is a bidirectional causal association between MDD and AD (53), and MDD can lead to a higher risk of CVDs (54). Therefore, the influence of potential pleiotropy remains a concern in this MR study, despite the lack of evidence from MR-Egger regression and MR-PRESSO methods. Third, due to the low variance explained by the SNPs and low sample size for some of the outcomes, the statistical power for the null associations may be insufficient. Thus, these negative results should be interpreted with caution. Finally, since the GWAS conducted by the EAGLE eczema consortium did not classify the severity of AD (12), we were not able to explore whether the risk of CVDs increases with increasing severity of AD, as several observational studies have suggested (6, 33, 56).

## Conclusions

The present MR study provided genetic evidence showing a causal association between genetic predisposition to AD and HF. Prevention and early diagnosis of AD may help prevent HF. No causal associations between AD and the risk of AF, CAD, MI, stroke, and stroke subtypes were found. Further studies are warranted to gain more insight into the underlying mechanisms.

## Data Availability Statement

The datasets presented in this study can be found in online repositories. The names of the repository/repositories and accession number(s) can be found in the article/Supplementary Material.

## Ethics Statement

The studies involving human participants were reviewed and approved by Local Ethics Committees of the UK Biobank project, CARDIoGRAMplusC4D, Nielsen et al. GWAS, HERMES consortium, the MEGASTROKE project, and the EAGLE eczema consortium. No additional ethical approval are required.

## Author Contributions

HC: study conception and design. HC and CZ: data analyses and draft preparation. LZ: supervision of the study. All authors contributed to the article and approved the submitted version.

## Funding

This work was supported by the National Natural Science Foundation of China [Grant Number 81873484], the Youth Program of National Natural Science Foundation of China [Grant Number 82000316], and the Nature Science Foundation of Zhejiang Province [Grant Number LZ16H020001].

## Conflict of Interest

The authors declare that the research was conducted in the absence of any commercial or financial relationships that could be construed as a potential conflict of interest.

## Publisher's Note

All claims expressed in this article are solely those of the authors and do not necessarily represent those of their affiliated organizations, or those of the publisher, the editors and the reviewers. Any product that may be evaluated in this article, or claim that may be made by its manufacturer, is not guaranteed or endorsed by the publisher.
